# Barriers and facilitators to implementation of the Free Water Protocol – findings from a national survey of acute stroke unit staff

**DOI:** 10.1186/s12913-026-14182-1

**Published:** 2026-02-25

**Authors:** Sabrina A. Eltringham, Nicola Martindale, Craig J. Smith, Sue Pownall, Elizabeth Lightbody

**Affiliations:** 1 Sheffield Hallam University, Sheffield, S1 1WB UK; 2 Sheffield Teaching Hospitals NHS Foundation Trust, Sheffield, S10 2JF UK; 3 Division of Cardiovascular Sciences, University of Manchester, Manchester, M13 9PT UK; 4 Manchester Centre for Clinical Neurosciences, Geoffrey Jefferson Brain Research Centre, Salford Royal Hospital, Northern Care Alliance NHS Trust, Manchester, M6 8HD UK; 5 University of Central Lancashire, Preston, PR1 2HE UK

**Keywords:** Deglutition, Deglutition disorders, Dysphagia, Acute stroke, Free Water Protocol

## Abstract

The Free Water Protocol (FWP) offers patients who are at risk of aspiration with thin fluids the option to drink water under controlled conditions to enhance quality of life and hydration while minimising respiratory complications. Research supports its use in selected patients in rehabilitation settings, however evidence in acute stroke is limited. This study aimed to investigate the barriers and facilitators to the FWP implementation from the perspectives of professionals working in acute stroke units in the United Kingdom to inform a future feasibility study. A national web-based survey was undertaken of acute stroke unit staff, including speech and language pathologists, nurses, doctors, dietitians, and support staff. The survey, informed by the Consolidated Framework for Implementation Research, explored perceived barriers and facilitators towards the FWP implementation. Responses (*N* = 171) highlighted key barriers such as concerns around risks of aspiration and chest infections, complexity of patient selection, training challenges, oral hygiene practices, high workload, staffing levels and high turnover of staff disrupting implementation, and lack of clinical guidelines and clear governance. Facilitators included supporting evidence, perceived patient benefits, strong multidisciplinary team (MDT) collaboration, leadership by senior clinicians, effective communication, and adaptability of the FWP specifically patient selection and delivery methods among the FWP users. Successful FWP implementation in acute stroke units depends on overcoming risk aversion, strengthening MDT working, enhancing education and training, and embedding robust mouthcare protocols. These findings will guide stakeholder workshops to codesign an implementation strategy for a feasibility study in an acute stroke unit setting.

**Supplementary Information:**

The online version contains supplementary material available at 10.1186/s12913-026-14182-1.

## Introduction

The Free Water Protocol (FWP) is a dysphagia management approach that gives people with suspected or confirmed aspiration on thin liquids, who are receiving fluids enterally or in thickened consistency, the option to drink plain water [1]. There are clear guidelines to minimise the risk of respiratory complications including strict eligibility criteria, thorough mouthcare, individualised strategies to optimise swallowing safety, close clinical monitoring and multidisciplinary working. The aim of the FWP is to improve quality of life, respect preferences for care, and promote normalisation, which can be restricted in the acute hospital setting [2, 3]. Perceived benefits include improved mood, faster recovery of swallowing function, better oral hygiene and comfort, and to a lesser degree hydration due to the small amounts consumed [2].

The Free Water Protocol was initially designed and implemented in rehabilitation settings, where patients are generally more medically stable and have longer lengths of stay. Acute populations, such as those in acute stroke units, may differ in levels of mobility, independence, and rehabilitation tolerance, which may increase susceptibility to respiratory complications. However, research mainly from rehabilitation studies, including stroke patients, has shown no deterioration in chest status in carefully selected patients and a trend towards improved hydration and patient satisfaction [4]. A recent Royal College of Speech and Language Therapists (RCSLT) position paper on the potential burdens and benefits of thickened fluids has raised the profile of the FWP as an alternative management approach for patients at risk of aspiration who dislike modified fluids [5]. The National Institute for Clinical Excellence (NICE) has recommended further research into use in stroke rehabilitation stating that current research is promising but limited [6].

A systematic review, conducted as part of this research programme, explored barriers and facilitators to the FWP implementation in acute stroke [7]. The review identified several barriers. These included a limited evidence base and the absence of a standard protocol. Adapting the FWP to the acute setting was challenging, as were patient selection and the complexity of the FWP design. A culture of risk aversion also made implementation more difficult. Staffing issues were another barrier, particularly reliance on agency nurses, a transient workforce, and the specialist skills required for FWP delivery. The review also identified important facilitators. National recommendations encouraged further research, while oral care protocols laid the groundwork for FWP implementation. The acute stroke setting itself offered unique opportunities to support the approach. Senior clinicians provided leadership and acted as role models. Interdisciplinary collaboration, with clear accountability, helped support the process. Regular communication and ongoing education were other facilitators along with involving patients in decision-making. Limitations of the review were the small number of studies retrieved, their heterogeneity with multiple studies from the same research laboratory.

This national survey aimed to investigate the barriers and facilitators of implementation of the FWP from the perspectives of acute stroke healthcare professionals. Insights from the survey and subsequent focus groups will inform stakeholder workshops, which will codesign an implementation strategy for a feasibility study of the implementation of the FWP for stroke survivors with dysphagia in the acute stroke unit setting.

## Methods

### Study design

A national cross sectional web-based survey of Speech Language Pathologists (SLPs), nurses, doctors, dietitians and clinical support staff was undertaken. The CHERRIES checklist [8] was used to ensure complete description of the methodology (Supplementary Material 1).

The survey questions were developed from a systematic review [7] and were underpinned by the Consolidated Framework for Implementation Research (CFIR) [9]. The CFIR is an implementation science framework designed to systematically identify and understand factors that influence the implementation of complex interventions in real‑world settings. It provides a menu of constructs arranged across five key domains: intervention characteristics, outer setting, inner setting, characteristics of individuals, and implementation process. In the context of a feasibility study, the CFIR supports the identification of determinants that may affect acceptability, adoption, and delivery of an intervention. By explicitly linking these determinants to potential implementation strategies, CFIR enables the purposeful design of strategies tailored to the local context.

The total number of survey items were 69. A branching mechanism was used where the questions presented were based on the respondent’s answers to previous questions. Questions were grouped by CFIR domains. (Supplementary Material 2). The survey was pretested before fielding to the target population. This involved a four-stage process; usability and technical functionality of the electronic questionnaire (Stage 1); pilot phase (Stage 2) where a member of the research team, two SLPs and a nurse completed the survey and responded to eight debriefing questions; final refinement based on the pilot feedback was carried out (Stage 3) before fielding the questionnaire to the target population (Stage 4).

### Survey administration

The sampling frame was National Health Service (NHS) staff with at least 6 months experience working on an Acute Stroke Unit. A convenience sampling method was used. The survey was advertised through social media and emailed to professional networks (Supplementary Material 3). Survey responses were captured automatically on the Qualtrics survey platform. The survey was open from 3/10/2024 until 7/11/2024. Only completed surveys were included.

### Analysis

Completed survey responses were exported to SPSS for Windows (Version 28.0). Descriptive statistics were used to describe and summarise categorical data. Several questions offered an ‘other’ response, and respondents were given the option to provide free text. An inductive approach was used allowing data segments to be labelled, coded and grouped into categories. The updated CFIR interview coding guidelines [10] were used to ensure fidelity with which data was mapped onto the appropriate domain. A second member of the research team (NM) checked the data mapping for trustworthiness.

## Results

### Participant characteristics

**Fig. 1:**
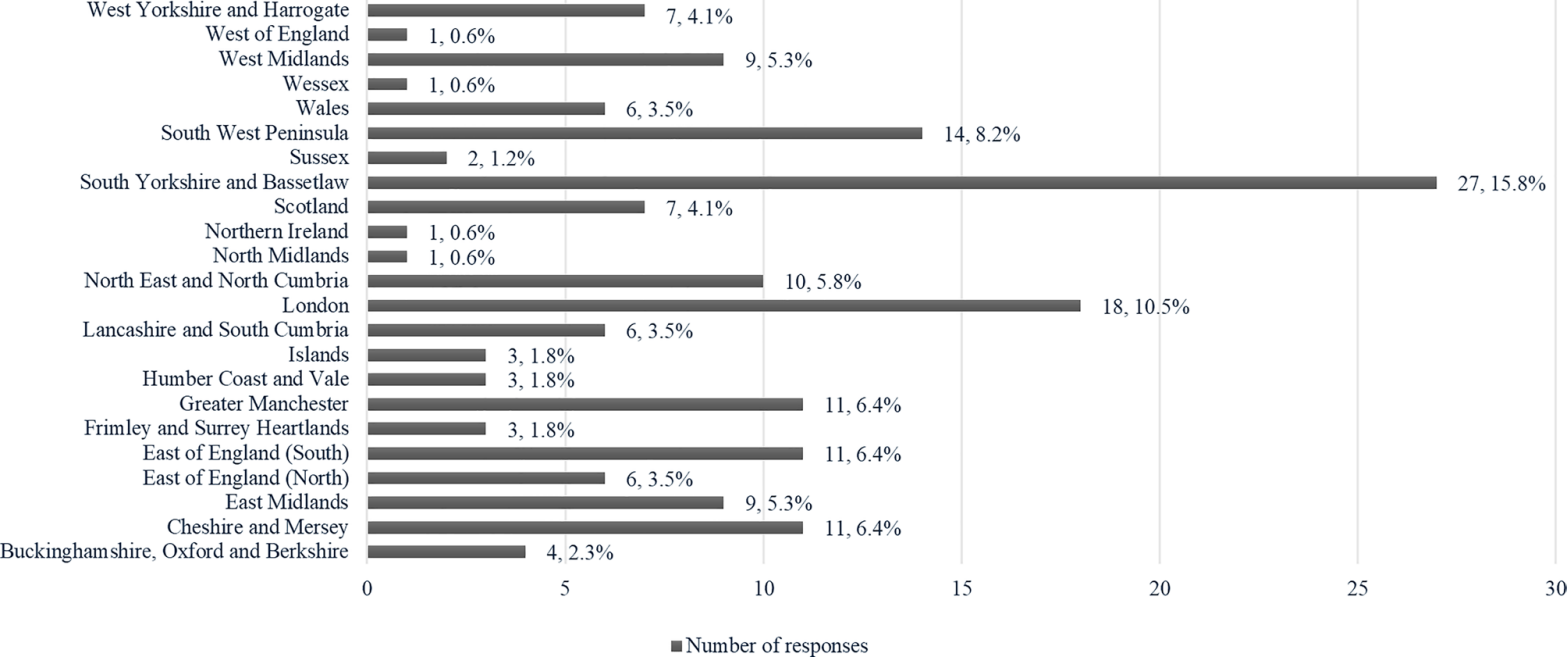
Responses by region

A total of *N* = 171 surveys were completed. Completion rate (ratio agreed to participate/finished survey) was 89.5%. Of the respondents, 64 (37.4%) used the Free Water Protocol (FWP), while 107 (62.6%) did not. The majority of responses were from SLPs (68.4%, *n* = 117), followed by nursing staff (17%, *n* = 29), dietitians (8.8%, *n* = 15), doctors (5.3%, *n* = 9), and clinical support staff (0.6%, *n* = 1). The number of participants in each discipline, stratified by users and non-users, is shown in Table 1. There was representation across the Integrated Stroke Delivery Network Regions in England, Wales, Scotland, and Northern Ireland (Fig. 1).

**Table 1:** Participant distribution by discipline and FWP use

Discipline	Users	%	Non Users	%
Nurse	8	13%	21	20%
Dietitian	2	3%	13	12%
SLP	50	78%	67	63%
Doctor	3	5%	6	6%
CSS	1	2%	0	0%
Total	64		107	

The findings are presented in alignment with the identified CFIR domains with quotations from respondents and summarised in Table 2.

#### Domain 1: Innovation -

This domain relates to the characteristics of the FWP as the intervention being implemented.

Both users and non-users of the FWP identified various sources of evidence supporting its use, most commonly published research (*n* = 110), clinical experience (*n* = 81) and national clinical guidelines (*n* = 55). Additional sources included anecdotal studies, pilot projects, presentations (conference/webinars), and courses (theoretical foundations such as McNeill dysphagia therapy). Thirty-six respondents were unsure of the evidence base. More FWP users felt this evidence facilitated implementation than hindered it. However, gaps in national guidelines, large-scale research, and safety criteria created uncertainty, especially for patients with severe dysphagia. Respondents highlighted the need for more rigorous studies, particularly in the acute stroke setting that addressed uncertainty around mobility and chest status; *‘I feel there needs to be more high quality evidence in acute stroke with acute stroke patients showing no adverse chest deterioration before full implementation’ [SLP non-user].*

Patient satisfaction (96.5%, *n* = 165) and choice (94.2%, *n* = 161) were the top-rated benefits of FWP, followed by hydration status (88.3%, *n* = 151), early swallow rehabilitation (81.3%, *n* = 139), and quicker diet or fluid upgrades (59.6%, *n* = 102). Additional benefits included better oral hygiene, enhanced comfort, and an overall improved quality of life. For patient outcomes: chest status and medical status, were mostly seen as neutral. Notably, only 4.7% of FWP users rated chest status as ‘disadvantageous,’ compared to 41.1% of non-users. One respondent stated, *‘Medical status trumps patient preference in terms of rehabilitation’[SLP non-user]*.

Identifying suitable patients was seen as complex by 53% of non FWP users versus 40.7% of users. Common barriers included: reduced alertness (85.3%, *n* = 146), respiration status (80.7%, *n* = 138), significant pharyngeal problems (80.1%, *n* = 137), reduced cognition (77.8%, *n* = 133) with one respondent commenting *‘It’s easier to implement if the pt is aware of the recs and can advocate for themselves’ [SLP user]*, significant oral swallowing problems (76.6%, *n* = 131), and poor secretion management (74.3%, *n* = 127). Other concerns were impulsivity (69.6%, *n* = 119) (although one respondent commented that ‘*impulsivity can be managed with support from others’ [SLP user]*), delirium (67.3%, *n* = 115), fatigue (56.7%, *n* = 97), poor mobility (41.5%, *n* = 71), patient is on a fluid restriction (41.5%, *n* = 71) and short hospital stays (12.3%, *n* = 21). Poor oral hygiene ‘*status of oral cavity and limitations of mouthcare access’ [SLP user]* and limited access to instrumental assessment were also noted. One respondent added: *‘The FWP is used very infrequently in our service. This is because not many patients meet the criteria… If a trial showed it was safe to implement with more dependent*,* less mobile*,* more cognitively impaired*,* more unwell patients then we would implement it more widely’ [SLP user]*.

#### Domain II: Outer Setting -

This domain captures external contextual factors influencing implementation, including the wider hospital environment and national policy context.

32% of users and non-users of the FWP (*n* = 54) felt conditions outside of the Acute Stroke Unit would positively affect FWP implementation, while 30% (*n* = 51) disagreed and 39% (*n* = 66) were unsure. Facilitators for implementation included integrated into stroke guidelines, advice or guidance from acute stroke units that have successfully implemented the FWP programme and available training and quick links to the evidence base and the RCSLT Position Paper [5] about ‘*reducing use of thickener*’ *[SLP non-user].* One respondent commented that *‘Governance have to clear changes being made in clinical patient care delivery’ [Nurse user].* Governance was also cited as a barrier to implementation. Other external barriers included bed pressures resulting in stroke patients being cared for on outlying and unfamiliar wards which was problematic for staff education and policy and procedures not being in place to allow for the protocol to be trialled. Transfer of patients from another unit where they may be using a different protocol was identified as a potential barrier; *the protocols would need to be similar across setting to make the most of the FWP’ [SLP non-user].*

#### Domain III: Inner Setting -

This domain refers to the organisational context of the Acute Stroke Unit within a UK NHS hospital where the intervention is implemented.

Mixed acute and rehabilitation wards were identified as a potential barrier; *‘there can be a lack of understanding of acute stroke management from support staff at times’ [SLP non-user]*, as were patients on non-stroke wards due to inconsistent staff understanding *‘stroke patients are often outlied to non-stroke wards which would likely then mean FWP not appropriate to continue - this would then feel difficult to manage if they had been “allowed” to have something on ASU but then not when moving to another ward’ [SLP non-user]*. Resources to support FWP capacity assessment were identified as tangible materials to facilitate its use. Barriers included lack of easy and quick access to instrumental swallowing assessments such as videofluoroscopy (VFS) and fibre optic endoscopic evaluation of swallowing (FEES).

Heavy workload (84.8%, *n* = 145), frequent monitoring (81.3%, *n* = 139), time intensity of stroke care (80.7%, *n* = 138) were major barriers, *‘Fast paced highly pressured ward and at times there is no time for intervention only assessment and management’ [SLP user].* Staff turnover (72.5%, *n* = 124), low staffing levels, consistency of staffing, limited stroke specialist nurses, and language barriers of international nurses further complicated implementation. One respondent commented *‘Appropriate staffing levels could ensure this protocol is carried out safely however at present staffing is too low to provide fundamentals of care never mind an additional risky protocol’ [Nurse non-user].* Another commented how staffing levels would greatly impact facilitation; *‘If we had better staffing*,* especially across SLT and nursing*,* I would feel more confident in facilitating it with our complex patient cohort’ [SLP non-user].*

Cohesiveness of the stroke multidisciplinary team (81.2%, *n* = 139), being part of the stroke MDT (77.2%, *n* = 132) and relationships with stroke unit colleagues (71.2%, *n* = 123) positively influenced implementation; *‘Good relationships inspire effective communication key to any implementation’ [Dietitian user]*. Engagement was identified as important and ‘*trust that it would do no harm compared to usual practice*’*[Dietitian non-user].* Other respondents referred to ‘*buy in*’*[SLP non-user]* from the nursing and medical teams and felt they would be supportive if fully informed of the potential benefits and if they were given clear guidance of their role in FWP implementation. Fewer respondents ‘somewhat agreed’ or ‘strongly agreed’ that relationships with colleagues in other areas of the hospital (55%, *n* = 94) were felt to affect implementation.

Miscommunication with the MDT about the recommendations was perceived as a barrier especially when the patient could not advocate for themselves. Most useful methods for sharing FWP included verbal communication at daily MDT meetings (61.4%, *n* = 105), verbal communication at weekly MDT meetings (49.1%, *n* = 84), electronic or paper handovers (35.1%, *n* = 60) and medical notes (34.5%, *n* = 59). Smaller numbers of participants selected methods like word of mouth, webinars, email or hospital intranet. Other methods for sharing information included: bedside signs, safety huddles, notice boards, Microsoft teams, screen savers, training sessions and staff appraisals.

Clinicians’ risk aversion (88.9%, *n* = 152) and prior experience (69.0%, *n* = 118) influenced decision making. One respondent identified *‘Our biggest barrier is SLT team being risk adverse’ [Nurse non-user]* another identified ‘*perceived associated risks’ [SLP non-user]* regarding MDT uptake. *‘I feel staff would not feel comfortable to go against SLT recommendations between meals’ [Dietitian non-user]*. Beliefs (68.4%, *n* = 117) and expectations of learning new processes and procedures (59.1%, *n* = 101) also played a role. Other examples of cultural factors included *‘breaking down the medical model where consultant leads all decisions. Where an MDT listens and learns from each other’ [SLP user]* and *‘clinicians’ confidence in medical teams monitoring of chest’ [SLP user]* One respondent commented about *‘ward staff feeling anxious and responsible for delivering this if the patient deteriorates’ [SLP user].*

SLPs (67.5%, *n* = 79) and Dietitians (66.7%, *n* = 10) largely disagreed that the FWP is basic nursing care. In contrast nurses were more evenly split (50:50) whereas doctors were more inclined to agree, with twice as many respondents agreeing with the statement (*N* = 6) compared to those that did not (*N* = 3). Key barriers included nurses’ availability (55.6%, *n* = 95), caseload demands (39.8%, *n* = 68), delegation of tasks for dependent patients (32.2%, *n* = 55), high turnover of patients (19.3%, *n* = 33), limited resources to record fluid intake (18.1%, *n* = 31) and fast pace of setting (17%, *n* = 29). Poor oral care provision and access to mouthcare had the potential to impact on the level of fit with existing work processes with one respondent commenting: *‘I do have concerns about the steps required to get consistent mouthcare procedures and documentation*,* and implementation of a standard procedure for FWP given difficulties we encounter with more general swallowing recommendations’[SLP].* 80% of all respondents (80.1%, *n =* 137) felt other duties were or would be prioritised over providing the FWP.

#### Training

With exception of 7 FWP users (4.1%) who indicated ‘none of the above’, FWP and non FWP users indicated dietitians, doctors, families and carers, nursing staff, SLPs, and support workers receive training or should be trained. Other groups identified included: advanced clinical practitioners (ACPs), therapy teams (physiotherapy occupational therapy, therapy assistants), nutritional assistants, student nurses, pharmacy, catering, housekeeper, hostesses, catering, domestic staff. One respondent stated, *‘all members of staff involved with any patient care’ [SLP non-user]*. Another identified the importance of *‘Explaining to patients and relatives why only certain patients are receiving this treatment/care and why them or their relatives are not able to have this’ [Nurse non-user].* For respondents using the FWP training was predominantly provided by SLPs (82%) followed by nursing staff (5%). Non FWP users indicated SLPs (60%) should provide training, followed by Stroke Nurse Educators (26%), nursing staff (10%) and other responses (4%) included: ACPs, dietitians, stroke nursing team, and ‘all staff/suitable relatives.’ One respondent stated, *‘My initial reactions was that Speech and Language Therapists would be best placed to provide this training however on reflection*,* if a key barrier is found to be nurses perception that this isn’t their role to deliver FWP then the training would be most effective if delivered by the senior nursing leaders to the nursing teams’ [SLP non-user].*

Suggested training formats varied: face-to-face teaching (FWP users 29% vs. 18% Non FWP users), individual face-to-face teaching (FWP 19% vs. Non FWP 8%), practical demonstration (FWP users 15% vs. Non FWP 9%), peer support and modelling (FWP 13% vs. Non FWP 12%) and written handouts (FWP 13% vs. Non FWP 11%). Less frequently selected formats included e-learning modules (such as in house oral care training, Mouthcare matters) (FWP 7% vs. Non FWP 14%), competency check (FWP 4% vs. Non FWP 12%) and Teach back (FWP 1% vs. Non FWP 9%). One FWP user stated that they were *‘Planning to implement 10-minute bite size sessions at nursing handover’ [SLP user]* and a non FWP user felt *‘Practice 1:1 training is likely to be most effective as this is active involving the persons full participation. From personal experience and delivering training e-learning tends to be clicked through and perceived as something that has to be completed to meet a trust requirement*’*[SLP non-user]*.

FWP users and non-users identified several barriers to training within Acute Stroke Units. The most frequently cited included time to deliver ongoing training (80.7%, *n* = 138) with one respondent stating, *‘Main barrier would be the time needed to implement FWP and to continue the education and support needed for this’ [SLP non-user]*, ongoing training requirements due to shift changes (71.9%, *n* = 123), organisation effort required to sustain training (61.4%, *n* = 105) and no clear written protocol (57.9%, *n* = 99). Eight FWP users felt ‘none of the above’ applied to their stroke unit. Additional challenges included inconsistent staffing (e.g. bank staff) and limited capacity to deliver training alongside clinical duties. One respondent stated *‘The key to its success on the unit is having experienced clinicians with positive experiences of FWP/selecting patients and regular MDT training and exposure so that FWP is a commonplace recommendations. This builds confidence among SLTs and the ward’ [SLP user].*

##### Domain IV: Individual characteristics -

This domain relates to the roles, beliefs, skills, and attributes of the individuals involved in the implementation of the FWP.

#### Roles

Most respondents (90.1%) felt the FWP aligned with their professional role, though dietitians (66.7%) were less likely to agree compared to other groups: SLPs (94.9%), doctors (88.8%), nurses (82.8%). The one support staff respondent felt it did fit within their role. Family involvement was common, particularly in offering water (*n* = 54), supervision while drinking (*n* = 51), mouth care (*n* = 40) and positioning (*n* = 32). Other support included ‘*alertness*,* wellness*,* reducing distractions*’ *[SLP user]* and ‘*supporting the patients to understand and recall the recommendations’ [SLP user].* Non users of the FWP added that involvement of families and caregivers would be dependent on the individual caregiver’s knowledge and skills and willingness to provide this support. One respondent stated, *‘With correct education*,* families/informal carers could and should support all aspects of FWP’ [SLP non-user]* however concerns were raised about inconsistent messaging and protocol adherence. Motivation to engage with the FWP was driven by patient choice (88.9%), patient desire to drink water (87.1%) and nurses desire to make time for the FWP (62%). Reported barriers included negative staff attitudes around completing mouthcare (61.4%), SLP uncertainty that the FWP would be implemented as intended (53.8%), legal concerns (41.5%) and time constraints for SLP (39.2%).

#### Beliefs, skills and attributes

Consultant approval and leadership from confident, experienced SLPs were seen as critical to successful implementation: *‘We only implement FWP which SLT have identified and had a discussion with the consultant’[SLP user].‘I think the lead SLT on ASU needs to have confidence in using the FWP and their approach to swallow rehabilitation needs to align with this i.e.*,* not being completely risk adverse*,* as this gives the whole SLT team the confidence to implement it and also the nursing team’ [SLP user].* The involvement of nutritional assistants and catering staff, alongside engagement with visitors and volunteers, was identified as supporting implementation. Pharmacy services were also highlighted as providing *‘excellent support’ [SLP user]*. Others identified family members as beneficial, though concerns were raised about misinterpretation of the protocol by relatives particularly understanding that *‘L0 water is not tea/squash etc’ [SLP non-user].*

**Fig. 2:**
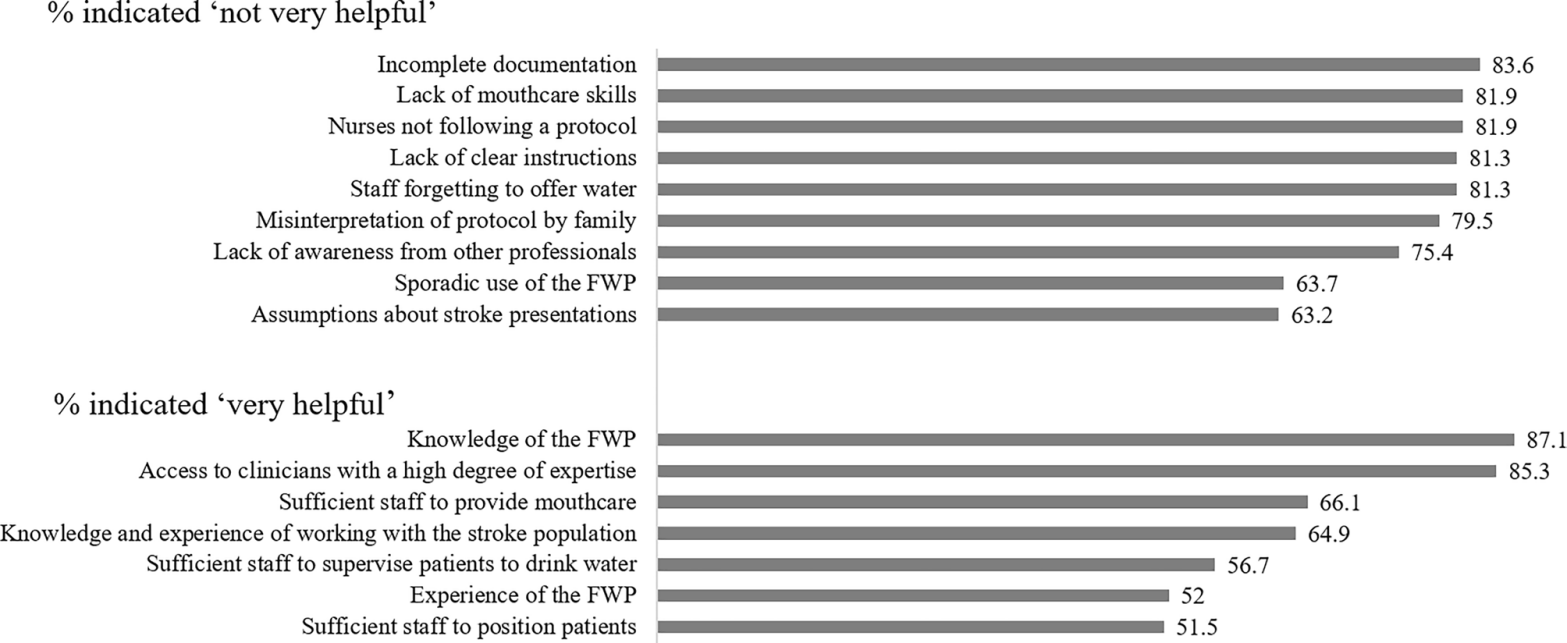
Factors relating to the MDT capability that influence FWP success

FWP and non FWP users strongly agreed that the FWP met patient needs by facilitating choice (70.2%), comfort (63.7%), hydration (59.1%) and normalisation (55.6%). One respondent stated that compared to usual care the FWP was ‘*feeling of not being ignored’ [Nurse non-user].* Other patient outcomes which were cited as facilitators to implementing the FWP were improved oral hygiene, mood and quality of life, and secretion management. However, concerns about negative patient outcomes including chest complications (50.9%) and aspiration (39.2%) were prominent, and most respondents (88%) felt that the potential for negative outcomes outweigh the potential positive outcomes. Other potential outcomes that were perceived as barriers to implementation were: any change in medical status post implementation, aspiration or penetration *‘if this causes discomfort’ [SLP non-user]*, impact on health outcomes and flow *‘if mismanaged in an acutely unwell patient with lower tolerance of infection’ [SLP non-user]* and reduced rehabilitation engagement. The extent that individual factors were felt to be help deliver the FWP are shown in Fig. 2.

##### V. Implementation Process -

This domain relates to the activities and strategies used to plan, deliver, and embed the FWP.

Percentages reported represent the proportion of FWP users who perceived each element as *“very important.”*

**Fig. 3:**
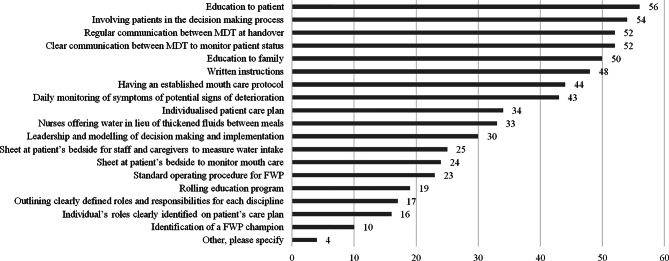
Strategies employed by FWP users to implement the FWP ranked by number of responses

Effective implementation was underpinned by intentional team collaboration regarding communication (87.5%), patient monitoring (84.4%), and patient selection (65.6%). Leadership by senior clinicians (65.6%), dedicated implementations leaders and champions (37.5%) and availability of family support (26.6%) were important to support team coordination. Suboptimal team collaboration (e.g. nursing perceptions of FWP as an SLP responsibility rather than their role) was perceived as a barrier to implementation. A range of strategies were employed by FWP users (Fig. 3). Over half of respondents indicated they provided patient and family education, involved patients in the decision making process and had regular communication between the MDT at handover. Identification of a FWP champion was least frequently used. Strategies included in the ‘other’ category were documentation of fluid intake and mouthcare on electronic case records and including the FWP as a recommendation on the patient’s drug chart.

Among non-users, the most frequently endorsed strategies mirrored those of users, with emphasis on MDT communication, education, and mouthcare protocols. Clear documentation of fluid intake and mouthcare was also highlighted as essential.

A consistent barrier to FWP implementation was the lack of established, high-quality oral care procedures *‘Oral care continues to be an issue and would pose greater risks to our patients unless it was in place correctly to begin with’ [SLP non user]*,* ‘We don’t have a formal FWP as we have continually struggled to establish an adequate oral hygiene baseline despite multiple hospital wide projects’ [SLP user]*,* ‘My biggest perceived barrier is the lack of routine effective mouthcare*,* I feel strongly this should be part of the implementation of FWP and a prerequisite’ [SLP non-user].* Key elements identified for inclusion in a FWP care plan were: level of supervision required (*n* = 158), documentation of mouthcare (*n* = 157), water offered (*n* = 154), patient or consultee consent (*n* = 147), accountability for mouthcare (*n* = 133), individuals’ fluid requirements (*n* = 130), evidence of education for staff (*n* = 128), evidence of education for family (*n* = 128). Two respondents identified chest status as important and 2 indicated ‘none of the above’.

Respondents reported both successful and unsuccessful trials. While some carried out a pilot project and developed clinical guidelines to implement the FWP, others discontinued use due to adverse outcomes such as chest infections. One “*trialled the FWP a couple of times but unfortunately on both occasions patients developed chest infections” [SLP non-user].*

Over half of users *(54.7%*,* n* = 35) adapted the protocol, only 18.7% did not. Seventeen users (26.6%) did not know if the FWP had been adapted. Ways that the protocol was adapted included patient selection (*n* = 21), water volume (*n* = 20) and mode of delivery (*n* = 20). Some offered ice chips as an alternative (*n* = 11). Some users said the amount offered (free sips, teaspoons only, capped amounts e.g., 5 teaspoons an hour) was adapted on an individual basis, with considerations for patient cognition, chest status, and mobility. Non users stated, “*Sometimes I may recommend teaspoons of water but is usually a restricted amount and restricted bolus size and following a VFS or FEES” [SLP non-user].* Another provided the FWP in an *“informal manner” [SLP non-user]* to those they felt would benefit but *“did not follow a strict protocol”* and another described using a “*water protocol rather than the FP” [SLP non-user].*

**Table 2:** Summary of the barriers and facilitators mapped to the CFIR domains. Results are presented as a summary across the two cohorts as findings were similar

I. INNOVATION	Barriers	Facilitators
B. Innovation Evidence-Base	Lack of robust evidence in acute stroke Lack of clinical guidelines	Published research articles Previous clinical experience
C. Innovation Relative Advantage	Chest status Medical status	Patient satisfaction and choice Hydration status Early swallow rehabilitation Oral hygiene and comfort QOL
D. Innovation Adaptability	Too restrictive criteria Countering the intent of the FWP e.g. offering ice chips instead of water, not offering water to people who aspirate	Amount of water offered Mode of delivery Patient selection criteria
E. Innovation Trialability	Patient factors: alertness, respiration status, significant pharyngeal problems, reduced cognition, significant oral problems, poor secretion management, impulsivity, delirium, fatigue, poor mobility, fluid restriction, Poor oral hygiene	-
F. Innovation Complexity	Patient selection	-
II. OUTER SETTING	**--**	**-**
C. Local Conditions	Bed pressures resulting in stroke patients on outlying wards	-
D. Partnerships and Connections	Patients on different protocols transferred to ASU	Guidance and resources from successful ASUs
E. Policies & Laws	Governance clearance No policies in place	RCSLT position paper Integration into Stroke Policy
III. INNER SETTING		
A. Structural characteristics	Access to instrumental assessments Speciality of care: mixed acute and rehab wards, stroke patients in non-specialist wards Staffing: Staffing levels, turnover, consistency, EAL,	Resources to support MCA
B. Relational Connections		Cohesiveness of the stroke MDT Inclusion in the stroke MDT Relationships with stroke unit colleagues Relationships with colleagues in other areas of the hospital Buy in from nursing and medical teams
C. Communications	Miscommunication within the MDT	Verbal communication at daily/ weekly handovers
D. Culture (deliverer and learning centeredness)	Clinicians attitude to risk Anxiety Breaking with established practices Perception of professional remit Previous experiences influencing decision making	Previous experiences influencing decision making Clinician’s beliefs influencing decision making Clinician’s expectations of learning new processes
F. Compatibility	Basic nursing care Nurses’ availability Poor oral care provision Caseload demands Delegation of tasks for dependent patients	-
G. Relative Priority	Prioritisation of other duties over the FWP	-
K. Access to Knowledge & Information	Training: Organisation, ongoing commitment and organisation. No clear written protocol	Education for staff: ACPs, dietitians, doctors, housekeepers, pharmacy, nurses, OTs, PTs, SLTs, student nurses, support staff, therapy and nutritional assistants, and informal caregivers
IV. INDIVIDUALS CHARACTERISTICS		
A. Need	Perceived negative outcomes: development of chest complications, aspiration	Perceived benefits: patient choice, comfort, hydration, normalisation, oral hygiene, mood, QOL and secretion management
B. Capability	Incomplete documentation, lack of mouthcare skills, nurses not following a protocol, lack of clear instructions, staff forgetting to offer water, misinterpretation of protocol by family, lack of FWP awareness from other professionals, sporadic use of the FWP and assumptions about stroke presentations	Knowledge and experience of the FWP and stroke population Resource capability: high degree of dysphagia expertise, sufficient staff to provide mouthcare, supervise and position patients,
D. Motivation	Attitudes towards mouthcare SLT uncertainty that the FWP would be implemented as intended	Patient choice and desire Nurses desire to make time
V. IMPLEMENTATION PROCESS		
A. Teaming	-	Intentional team collaboration: communication, support to identify and monitor patients Leadership by senior clinicians
E. Tailoring Strategies	-	Patient and family engagement and education: Involving patients in decision making, education to patients, education to family members, providing written instructions MDT communication and coordination: Clear MDT communication to monitor patient status, regular MDT communication at handover, nurses offering water in lieu of thickened fluids between meals, leadership and modelling of decision-making and implementation, identification of an FWP champion Clinical Monitoring & Safety Measures: Established mouthcare protocol prior to FWP implementation, daily monitoring for signs of deterioration associated with aspiration, bedside sheet to measure water intake, bedside sheet to monitor mouthcare, recording fluid intake and mouthcare in electronic case records Individualised Care Planning: Individualised FWP care plan, clearly defined roles and responsibilities for each discipline, everyone’s role identified on the patient’s care plan, FWP listed as a recommendation on the drug chart Training, Education & Workforce Support: Rolling education programme for team members Standard Operating Procedure (SOP) for FWP
G. Doing	Unsuccessful trial with patients developing chest infections	Pilot project and clinical guidelines in place
I. Adapting	Patient selection, amount offered and mode of delivery.	

Key - QOL=Quality of Life, FWP=Free Water Protocol, ASU=Acute Stroke Unit, RCSLT=Royal College of Speech and Language Therapists, MCA=Mental Capacity Assessment, EAL = English as an Additional Language, MDT=Multidisciplinary Team, ACPs=Advanced Clinical Practitioners, OTs=Occupational Therapists, PTs=Physiotherapists, SLPs=Speech and Language Pathologists

## Discussion

Evidence supporting the Free Water Protocol (FWP) has largely been derived from rehabilitation settings, where studies suggest benefits for hydration, oral comfort, and quality of life without increased risk of aspiration pneumonia in carefully selected patients [4]. In contrast, evidence within acute stroke care remains limited, and uncertainties persist around patient selection, safety, and feasibility of delivery in high-intensity clinical environments [2, 3]. National guidelines reflect this position: the recent NICE guideline on stroke rehabilitation [5] and the RCSLT position paper on thickened fluids [6] recommend further research into its use and recognise the FWP as a potential alternative intervention when supported by robust mouthcare and safety measures. A recent systematic review further highlighted barriers to acute implementation including a limited evidence base, risk-averse culture, and staffing challenges alongside facilitators such as established oral care protocols, clinical leadership, and multidisciplinary collaboration [7]. This survey extends this evidence by exploring the perspectives of acute stroke unit staff across the UK.

Our survey data indicate the perceived benefits of the FWP, such as improved hydration, patient satisfaction, and quality of life were widely acknowledged, however implementation remains inconsistent due to concerns around safety, staffing, and infrastructure particularly in the absence of robust evidence from acute settings. Respondents highlighted gaps in high-quality, large‐cohort trials in acute stroke settings and definitive selection criteria, especially regarding mobility and chest status following stroke. This lack of conclusive evidence contributed to uncertainty and hesitancy, underscoring the need for rigorous research in acute stroke settings before widespread adoption can be confidently recommended.

Implementation was shaped by contextual factors both outside and within the stroke unit. Externally, governance processes, bed pressures, and potential differences in FWP protocols across hospitals were perceived as potential barriers. Internally, mixed ward layouts, limited access to instrumental swallowing assessments, high workloads compounded by staffing shortages and turnover created an environment in which FWP delivery had the potential to be deprioritised. A key finding was the critical role of oral care as a prerequisite for FWP implementation. Respondents consistently reported that inadequate mouthcare posed a significant risk, echoing previous studies that identified a high incidence of stroke-associated pneumonia in patients with poor oral hygiene.[11, 12] Similar concerns were raised by Gillman et al. [4], who emphasised that rigorous oral care is essential to mitigate risks associated with thin liquid aspiration. Conversely, cohesive multidisciplinary teamwork, clear roles and responsibilities, and regular verbal handover discussions emerged as powerful facilitators as evidenced in other implementation studies [13, 14]. These factors highlight the importance of fostering strong communication channels and shared ownership - core features of the original protocol [1] - and were identified as implementation approaches requiring further exploration to strengthen FWP fidelity in the acute stroke setting.

Training emerged as a critical enabler, with SLPs typically leading education efforts. However, broader involvement from nursing leadership and tailored, practice-based approaches may enhance protocol fidelity. Local adaptations such as modifying patient selection criteria, mode of delivery, and delivery methods to reconcile protocol guidelines with individual patient needs were common. These adaptations, together with strong confident leadership from SLP leads and designated FWP “champions,” were felt to be instrumental in embedding the protocol into routine care. This aligns with prior research suggesting that flexibility in FWP application is necessary to accommodate patient variability. However, the lack of standardisation may also contribute to inconsistent outcomes and clinician uncertainty.

Building on these findings we will conduct focus groups to explore how staffing pressures, turnover, and competing workloads influence FWP implementation, while gathering practical strategies to address workflow barriers. They will also explore how to strengthen team‑wide commitment to the protocol, recognising that although SLPs often initiate it, successful uptake relies on engagement across all staff groups. Concerns about risk remain a barrier despite evidence from rehabilitation⁴ and small critical‑care studies [15] suggesting the FWP can be delivered safely with appropriate safeguards; however, uncertainty persists in acute stroke settings where evidence is more limited.

In addition, participants will discuss the beliefs underlying clinicians’ reluctance, clarify implementation roles, and identify effective approaches to training, including who should teach different staff groups. Finally, the sessions will gather input on the design and use of an FWP care plan and what information it should contain and who should access it.

### Study limitations

It was not possible to estimate the response rate based on the unknown number of potential respondents eligible to complete the survey. An alternative response rate measure - survey completion rate - the accepted measure of attrition, was calculated. Risk of participant bias was minimised through distribution of the survey through multidisciplinary stroke networks. However, there were a limited response from clinical support staff compared to other professional groups. This may have been due to the perception of the FWP being the domain of other staff groups, difficulty reaching this group of staff due to lack of professional body, reliance on other staff to cascade the survey and access to the internet. This under-representation may have influenced the perspectives captured, as these staff are often closely involved in the practical implementation of the FWP and may experience different operational day-to-day barriers and facilitators. There may be also a risk of multiple responses from the same respondent however this was minimised by each response being hand checked for partially complete responses and if respondents provided their contact details to be sent the results.

## Conclusion

This national survey of NHS acute stroke unit staff highlights the challenges of implementing the FWP in an acute stroke unit. While clinicians recognise the potential benefits for patient choice, quality of life, and rehabilitation, significant barriers remain, namely perceptions of risk, staffing capacity, and oral care practices. Addressing these barriers through robust training, interprofessional collaboration, leadership engagement, and embedding oral care protocols will be essential for success. Insights gained from this survey will directly inform stakeholder workshops and the co-development of an implementation strategy for a future feasibility study about the FWP in the acute stroke unit setting. If the future feasibility study can optimise facilitators and minimise barriers to achieve fidelity of the FWP in the acute stroke setting then evidence can be generated regarding the safety and effectiveness of the FWP with the acute stroke population.

## Supplementary Information

Below is the link to the electronic supplementary material.

Supplementary Material 1

## Data Availability

All data generated or analysed during this study are included in this published article and its supplementary information files.
