# Supplementary material for: Barriers and facilitators to implementation of the Free Water Protocol – findings from a national survey of acute stroke unit staff
Source: BMC Health Serv Res. 2026 Feb 25;26:443. doi: 10.1186/s12913-026-14182-1 (PMC13041077; doi:10.1186/s12913-026-14182-1)
Supplement: Supplementary file 1 — Supplementary Material 1 [file 12913_2026_14182_MOESM1_ESM.docx]

**SUPPLEMENTARY MATERIAL**

**Title:** Barriers and facilitators to implementation of the Free Water Protocol – Findings from a National Survey of Acute Stroke Unit staff.

**1: Checklist for Reporting Results of Internet E-Surveys (CHERRIES)**

| Item Category | Checklist Item | Explanation | Section/Page/line no(s). |
| --- | --- | --- | --- |
| Design | Describe survey design | Describe target population, sample frame. Is the sample a convenience sample? (In “open” surveys this is most likely.) | *See Methods section, sub heading Study design and data source.* |
| IRB (Institutional Review Board) approval and informed consent process | IRB approval | Mention whether the study has been approved by an IRB | *See Ethical approval and informed consent process.* |
|  | Informed consent | Describe the informed consent process. Where were the participants told the length of time of the survey, which data were stored and where and for how long, who the investigator was, and the purpose of the study? | *See Ethical approval and informed consent process.* |
|  | Data protection | If any personal information was collected or stored, describe what mechanisms were used to protect unauthorized access. | *The name and email address of respondents who chose to provide their information to be sent the results were stored on a password protected computer in the hospital computer network.* |
| Development and pre-testing | Development and pre-testing | State how the survey was developed, including whether the usability and technical functionality of the electronic questionnaire had been tested before fielding the questionnaire. | *Pretesting of the survey involved a four-stage process; usability and technical functionality of the electronic questionnaire (Stage 1); pilot phase (Stage 2) where a member of the research team, two SLPs and a nurse completed the survey and responded to eight debriefing questions; final refinement based on the pilot feedback was carried out (Stage 3) before fielding the questionnaire to the target population (Stage 4).* |
| Recruitment process and description of the sample having access to the questionnaire | Open survey versus closed survey | An “open survey” is a survey open for each visitor of a site, while a closed survey is only open to a sample which the investigator knows (password-protected survey). | *The electronic link to the survey was included in social media messages and in an email which was distributed through professional networks. Within the social media advert and email there was a description of participant eligibility. See Methods section, sub heading Study design and data source.* |
|  | Contact mode | Indicate whether or not the initial contact with the potential participants was made on the Internet. (Investigators may also send out questionnaires by mail and allow for Web- based data entry.) | *See Methods section, sub heading Survey administration.* |
|  | Advertising the survey | How/where was the survey announced or advertised? Some examples are offline media (newspapers), or online (mailing lists – If yes, which ones?) or banner ads (Where were these banner ads posted and what did they look like?). It is important to know the wording of the announcement as it will heavily influence who chooses to participate. Ideally the survey announcement should be published as an appendix. | *The survey was advertised through social media networks X and Linkedin and distributed through the research team and professional networks. Professional networks included the British and Irish Association of Stroke Physicians, British Dietetic Association, Integrated Stroke Delivery Network, Royal College of Speech and Language Therapists and National Stroke Nursing Forum. The social media advert is included in the supplementary media.* |
| Survey administration | Web/E-mail | State the type of e-survey (eg, one posted on a Web site, or one sent out through e-mail). If it is an e-mail survey, were the responses entered manually into a database, or was there an automatic method for capturing responses? | *See Methods section, sub heading Survey administration.* |
|  | Context | Describe the Web site (for mailing list/newsgroup) in which the survey was posted. What is the Web site about, who is visiting it, what are visitors normally looking for? Discuss to what degree the content of the Web site could pre-select the sample or influence the results. For example, a survey about vaccination on a anti-immunization Web site will have different results from a Web survey conducted on a government Web site | *See Methods section, sub heading Survey administration. The survey was not posted on a website. The hyperlink to the e-survey opened a browser to the survey webpage.* |
|  | Mandatory/voluntary | Was it a mandatory survey to be filled in by every visitor who wanted to enter the Web site, or was it a voluntary survey? | *See Ethical approval and informed consent process* |
|  | Incentives | Were any incentives offered (eg, monetary, prizes, or non-monetary incentives such as an offer to provide the survey results)? | *There was an offer to share the results of the results which was the penultimate question of the survey.* |
|  | Time/Date | In what timeframe were the data collected? | *See Methods section, sub heading Survey administration.* |
|  | Randomization of items or questionnaires | To prevent biases items can be randomized or alternated. | *Items were not alternated or randomised.* |
|  | Adaptive questioning | Use adaptive questioning (certain items, or only conditionally displayed based on responses to other items) to reduce number and complexity of the questions. | *Questions were hidden or revealed based on previously answered questions.* |
|  | Number of Items | What was the number of questionnaire items per page? The number of items is an important factor for the completion rate. | *The total number of items were 69.* |
|  | Number of screens (pages) | Over how many pages was the questionnaire distributed? The number of items is an important factor for the completion rate. | *Number of screens was dependent on question route.* |
|  | Completeness check | It is technically possible to do consistency or completeness checks before the questionnaire is submitted. Was this done, and if “yes”, how (usually JAVAScript)? An alternative is to check for completeness after the questionnaire has been submitted (and highlight mandatory items). If this has been done, it should be reported. All items should provide a non-response option such as “not applicable” or “rather not say”, and selection of one response option should be enforced. | *See Methods section, sub heading Study design and data source.* |
|  | Review step | State whether respondents were able to review and change their answers (eg, through a Back button or a Review step which displays a summary of the responses and asks the respondents if they are correct). | *Respondents were able to review and change their options through a ‘back’ button. The predicted duration of the survey was 15 minutes.* |
| Response rates | Unique site visitor | If you provide view rates or participation rates, you need to define how you determined a unique visitor. There are different techniques available, based on IP addresses or cookies or both. | *Not applicable* |
|  | View rate (Ratio unique site visitors/unique survey visitors) | Requires counting unique site visitors (not page views!) divided by the number of unique visitors of the first page of the survey. It is not unusual to have view rates of less than 0.1 % if the survey is voluntary. | *Not applicable* |
|  | Participation rate (Ratio unique survey page visitors/agreed to participate)  page (or agreed to participate). This can also be called “recruitment” rate. | Count the unique number of visitors who visit the first page of the survey (or the informed consents page, if present) divided by the number of people who filled in the first survey | *Not applicable* |
|  | Completion rate (Ratio agreed to participate/finished survey) | The number of people agreeing to participate (or submitting the first survey page) divided by the number of people submitting the last questionnaire page. This is only relevant if there is a separate “informed consent” page or if the survey goes over several pages. This is a measure for attrition. Note that “completion” can involve leaving questionnaire items blank. This is not a measure for how completely questionnaires were filled in. (If you need a measure for this, use the word “completeness rate”.) | *See Results - Completion rate was calculated as the number agreed to participate divided by number of finished surveys.* |
| Preventing multiple entries from the same individual | Cookies used | Indicate whether cookies were used to assign a unique user identifier to each client computer. If so, mention the page on which the cookie was set and read, and how long the cookie was valid. Were duplicate entries avoided by preventing users access to the survey twice; or were duplicate database entries having the same user ID eliminated before analysis? In the latter case, which entries were kept for analysis (eg, the first entry or the most recent)? | *Cookies were not used. There was a process for dealing with the possibility of multiple entries. See Discussion subheading Study Limitations section.* |
|  | IP check | Indicate whether the IP address of the client computer was used to identify potential duplicate entries from the same user. If so, mention the period of time for which no two entries from the same IP address were allowed (eg, 24 hours). Were duplicate entries avoided by preventing users with the same IP address access to the survey twice; or were duplicate database entries having the same IP address within a given period of time eliminated before analysis? If the latter, which entries were kept for analysis (eg, the first entry or the most recent)? | *IP addresses were deliberately not used. There was a process for dealing with the possibility of multiple entries. See Discussion subheading Study Limitations section.* |
|  | Log file analysis | Indicate whether other techniques to analyze the log file for identification of multiple entries were used. If so, please describe. | *Non applicable.* |
|  | Registration | In “closed” (non-open) surveys, users need to login first and it is easier to prevent duplicate entries from the same user. Describe how this was done. For example, was the survey never displayed a second time once the user had filled it in, or was the username stored together with the survey results and later eliminated? If the latter, which entries were kept for analysis (eg, the first entry or the most recent)? | *Non applicable.* |
| Analysis | Handling of incomplete questionnaires | Were only completed questionnaires analyzed? Were questionnaires which terminated early (where, for example, users did not go through all questionnaire pages) also analyzed? | *This is outlined in the Methods Section - only completed questionnaires were analysed.* |
|  | Questionnaires submitted with an atypical timestamp | Some investigators may measure the time people needed to fill in a questionnaire and exclude questionnaires that were submitted too soon. Specify the timeframe that was used as a cut-off point, and describe how this point was determined. | *Qualtrics measures the time people take to complete the survey and offers the facility for people to return to the survey if they take a break partway through. The estimated time duration of the survey calculated by Qualtrics was used to monitor an atypical timestamp.* |
|  | Statistical correction | Indicate whether any methods such as weighting of items or propensity scores have been used to adjust for the non- representative sample; if so, please describe the methods. | *Not applicable.* |

2: Published electronic version of the survey

Barriers and Facilitators to implementation of the Free Water Protocol in Acute Stroke

Start of Block: Introduction

Q1
**Barriers and Facilitators to implementation of the Free Water Protocol in Acute Stroke**   We would like to invite you to take part in a survey. The survey is about providing a treatment for stroke patients with swallowing problems known as the **Free Water Protocol**. The Free Water Protocol (FWP) gives patients who are recommended thickened fluids or nothing by mouth the option of drinking plain water between mealtimes. Patients follow guidelines to minimise the risk of adverse consequences and maximise patient safety.     We want to address some of the uncertainties about delivering the Free Water Protocol in an **Acute Stroke Unit** by better understanding the **barriers** and **facilitators** to implementation. We are interested in hearing from staff working in National Health Service (NHS) Acute Stroke Units that **DO** and **DO NOT** routinely use the FWP. For more information about the Free Water Protocol please look in the Appendix of the Participant Information Sheet .    This survey is relevant to you if you are a **nurse, clinical support staff, speech and language therapist, dietitian** or a **doctor** with at least 6 months NHS hospital Acute Stroke Unit experience.    Please read the Participant Information Sheet and consider whether you wish to take part. By completing and submitting the survey, you will be providing consent for the data to be included in the final analysis.     Thank you.

Q2 Are you a nurse, clinical support staff, speech and language therapist, dietitian or doctor working in a National Health Service (NHS) hospital Acute Stroke Unit?

- Yes. I confirm I am a nurse, clinical support staff, speech and language therapist, dietitian or doctor working in a National Health Service (NHS) hospital Acute Stroke Unit with at least 6 months Acute Stroke Unit experience. I have read the participation information sheet and give consent for the information I provide to be used for research purposes. (1)
- No. I am sorry you are not eligible to complete this survey. You will be re-directed to the final page of the survey. (2)

Skip To: End of Survey If Are you a nurse, clinical support staff, speech and language therapist, dietitian or doctor working = No. I am sorry you are not eligible to complete this survey. You will be re-directed to the final page of the survey.

End of Block: Introduction

Start of Block: Please indicate your clinical role

| 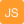 |
| --- |

Q3 Please indicate your clinical role.

- Clinical Support Staff (8)
- Dietitian (6)
- Doctor (9)
- Nurse (5)
- Speech and Language Therapist (7)

Q4 Please indicate how much experience you have working in an Acute Stroke Unit.

- 6 months to 2 years (1)
- 3 to 5 years (2)
- 6 to 10 years (3)
- 11 to 15 years (4)
- More than 15 years (5)

Q5 Please select your Integrated Stroke Delivery Network (ISDN) region.

- Buckinghamshire, Oxford and Berkshire (1)
- Cheshire and Mersey (2)
- East Midlands (3)
- East of England (North) (4)
- East of England (South) (5)
- Frimley and Surrey Heartlands (6)
- Greater Manchester (7)
- Humber Coast and Vale (8)
- Islands (9)
- Kent and Medway (10)
- Lancashire and South Cumbria (11)
- London (12)
- North East and North Cumbria (13)
- North Midlands (15)
- Northern Ireland (16)
- Scotland (17)
- South Yorkshire and Bassetlaw (18)
- Sussex (19)
- South West Peninsula (20)
- Wales (21)
- Wessex (22)
- West Midlands (23)
- West of England (24)
- West Yorkshire and Harrogate (25)

End of Block: Please indicate your clinical role

Start of Block: Do you use the Free Water Protocol (FWP) routinely in your Acute Stroke Unit?

Q6 Do you use the Free Water Protocol (FWP) in your Acute Stroke Unit?

- Yes (1)
- No (3)

End of Block: Do you use the Free Water Protocol (FWP) routinely in your Acute Stroke Unit?

Start of Block: FWP Intervention

Display This Question:

If Do you use the Free Water Protocol (FWP) in your Acute Stroke Unit? = Yes

| 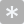 |
| --- |

Q7 In your opinion how does the FWP compare to **usual care** for the following patient outcomes?

|  | Advantageous (1) | Neither Advantageous or Disadvantageous (2) | Disadvantageous (3) |
| --- | --- | --- | --- |
| Patient satisfaction (2) |  |  |  |
| Chest status (4) |  |  |  |
| Early rehabilitation of swallow (5) |  |  |  |
| Quicker diet and/or fluid upgrade (6) |  |  |  |
| Hydration status (7) |  |  |  |
| Patient choice (8) |  |  |  |
| Medical status (9) |  |  |  |
| Other, please specify (10) |  |  |  |

Display This Question:

If Do you use the Free Water Protocol (FWP) in your Acute Stroke Unit? = No

| 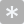 |
| --- |

Q8 In your opinion how would the FWP compare to **usual care** for the following patient outcomes?

|  | Advantageous (1) | Neither Advantageous or Disadvantageous (2) | Disadvantageous (3) |
| --- | --- | --- | --- |
| Patient Satisfaction (1) |  |  |  |
| Chest status (2) |  |  |  |
| Early rehabilitation of the swallow (3) |  |  |  |
| Quicker diet and/or fluid upgrade (4) |  |  |  |
| Hydration status (5) |  |  |  |
| Patient choice (6) |  |  |  |
| Medical status (7) |  |  |  |
| Other, please specify (8) |  |  |  |

| Page Break |  |
| --- | --- |

Display This Question:

If Do you use the Free Water Protocol (FWP) in your Acute Stroke Unit? = Yes

Q9 Has the FWP been **adapted** for use in your Acute Stroke Unit?

- Yes (4)
- No (6)
- Don't know (7)

Display This Question:

If Has the FWP been adapted for use in your Acute Stroke Unit? = Yes

Q10 **How** was the FWP **adapted**? Please select all that apply.

- Patient selection criteria (1)
- Amount of water offered (2)
- Offered ice chips as an alternative to water (3)
- Water was not offered to people who showed evidence of aspiration on Level 0 Thin Fluids on instrumental assessment (4)
- Mode of delivery e.g. from a teaspoon (6)
- Other, please specify (5) __________________________________________________

Display This Question:

If Has the FWP been adapted for use in your Acute Stroke Unit? != Yes

Q11 In your opinion **can** the FWP be **adapted** for use in the Acute Stroke Unit setting? *For more information about the Free Water Protocol please look in the Appendix of the Participant Information Sheet.*

- Yes (1)
- No (2)
- Don't know (3)

Display This Question:

If In your opinion can the FWP be adapted for use in the Acute Stroke Unit setting? For more informa... = No

Q12 What are the **barriers** to **adapting** the FWP to the Acute Stroke Unit setting?

- The complexity of the FWP intervention (1)
- The fact that the FWP was designed for the rehabilitation setting (2)
- Lack of agreement about how to implement the FWP (3)
- Mode of delivery e.g. from a teaspoon (5)
- Other, please specify (4) __________________________________________________

Display This Question:

If In your opinion can the FWP be adapted for use in the Acute Stroke Unit setting? For more informa... = Yes

Q13 In your opinion what **features** of the FWP are **adaptable**?

- Patient selection criteria (1)
- Amount of water offered (2)
- Offer ice chips instead of water (3)
- Water is not offered to people who aspirate Level 0 Thin Fluids on instrumental assessment (4)
- Other, please specify (5) __________________________________________________

Display This Question:

If Do you use the Free Water Protocol (FWP) in your Acute Stroke Unit? = Yes

Q14 How **complex** is it to identify which patients are **suitable** for the FWP? *This might include considering factors such as patient dependency, respiratory function, cognitive function, comorbidities, stroke diagnosis, aspiration status or patient choice.*

- Not complex at all (1)
- Slightly complex (2)
- Moderately complex (3)
- Very complex (4)

Display This Question:

If Do you use the Free Water Protocol (FWP) in your Acute Stroke Unit? = No

Q15 How **complex** would it be to identify which patients are **suitable** for the FWP? *This might include considering factors such as patient dependency, respiratory function, cognitive function, comorbidities, stroke diagnosis, aspiration status or patient choice.*

- Not complex at all (1)
- Slightly complex (2)
- Moderately complex (3)
- Very complex (4)

Q16 The following factors have been identified as **barriers** to **offering** the FWP to acute stroke patients. Which if any do you think are barriers?

- Poor mobility (1)
- Reduced cognition (2)
- Respiration status (3)
- Reduced alertness (4)
- Fatigue (5)
- Impulsivity (6)
- Delirium (7)
- Significant oral swallowing problems i.e. water falls out of the mouth (8)
- Significant pharyngeal swallowing problems i.e. no swallow reflex is triggered (9)
- Poor secretion management (10)
- Patient is a on fluid restriction (11)
- Short length of hospital stay (12)
- Other, please specify (13) __________________________________________________
- None of the above (52)

End of Block: FWP Intervention

Start of Block: Outer Setting

Display This Question:

If Do you use the Free Water Protocol (FWP) in your Acute Stroke Unit? = Yes

Q17 Were there any conditions **external** to your Acute Stroke Unit that **facilitated** the implementation of the FWP? *Conditions may include but are not limited to staffing, bed capacity, governance, economic, environmental, political or information technology (IT) systems.*

- Yes (1)
- No (2)
- Don't know (3)

Display This Question:

If Were there any conditions external to your Acute Stroke Unit that facilitated the implementation... = Yes

And Do you use the Free Water Protocol (FWP) in your Acute Stroke Unit? = Yes

Q18 Please tell us what these external conditions were and how they **facilitated** implementation.

________________________________________________________________

Display This Question:

If Do you use the Free Water Protocol (FWP) in your Acute Stroke Unit? = Yes

Q19 Were there any conditions **external** to your Acute Stroke Unit that acted as a **barrier** to implementation of the FWP? *Conditions may include but are not limited to staffing, bed capacity, governance, economic, environmental, political or information technology (IT) systems.*

- Yes (1)
- No (2)
- Don't know (3)

Display This Question:

If Were there any conditions external to your Acute Stroke Unit that acted as a barrier to implement... = Yes

And Do you use the Free Water Protocol (FWP) in your Acute Stroke Unit? = Yes

Q20 Please tell us what these external conditions were and how they acted as a **barrier** to implementation.

________________________________________________________________

Display This Question:

If Do you use the Free Water Protocol (FWP) in your Acute Stroke Unit? = No

Q21 Are there any conditions **outside** of your Acute Stroke Unit that would **facilitate** the implementation of the FWP? *Conditions may include but are not limited to staffing, bed capacity, governance, economic, environmental, political or information technology (IT) systems.*

- Yes (1)
- No (2)
- Don't know (3)

Display This Question:

If Do you use the Free Water Protocol (FWP) in your Acute Stroke Unit? = No

And Are there any conditions outside of your Acute Stroke Unit that would facilitate the implementati... = Yes

Q22 Please tell us what these conditions are and how they would **facilitate** the implementation of the FWP.

________________________________________________________________

Display This Question:

If Do you use the Free Water Protocol (FWP) in your Acute Stroke Unit? = No

Q23 Are there any conditions **outside** of your Acute Stroke Unit that would act as a **barrier** to the implementation of the FWP? *Conditions may include but are not limited to staffing, bed capacity, governance, economic, environmental, political or information technology (IT) systems.*

- Yes (1)
- No (2)
- Don't know (3)

Display This Question:

If Do you use the Free Water Protocol (FWP) in your Acute Stroke Unit? = No

And Are there any conditions outside of your Acute Stroke Unit that would act as a barrier to the imp... = Yes

Q24 Please tell us what these conditions are and how they would act as a **barrier** to implementation.

________________________________________________________________

Display This Question:

If Do you use the Free Water Protocol (FWP) in your Acute Stroke Unit? = Yes

Q25 When the FWP was introduced in your Acute Stroke Unit what were the **sources of evidence** for its use? Please select all that apply.

- National Clinical Guidelines (1)
- Published research articles (2)
- A Pilot Project (3)
- Previous clinical experience (4)
- Anecdotal stories from colleagues (5)
- Other, please specify (6) __________________________________________________
- None of the above (7)
- Don't know (8)

Display This Question:

If Do you use the Free Water Protocol (FWP) in your Acute Stroke Unit? = Yes

And When the FWP was introduced in your Acute Stroke Unit what were the sources of evidence for its u... != Don't know

And When the FWP was introduced in your Acute Stroke Unit what were the sources of evidence for its u... != None of the above

Q26 Did any of this evidence **facilitate** the implementation of the FWP in your Acute Stroke Unit?

- Yes (1)
- No (2)

Display This Question:

If Do you use the Free Water Protocol (FWP) in your Acute Stroke Unit? = Yes

And When the FWP was introduced in your Acute Stroke Unit what were the sources of evidence for its u... != None of the above

And When the FWP was introduced in your Acute Stroke Unit what were the sources of evidence for its u... != Don't know

Q27 Did any of this evidence act as a **barrier** to the implementation of the FWP in your Acute Stroke Unit?

- Yes (1)
- No (2)

Display This Question:

If Do you use the Free Water Protocol (FWP) in your Acute Stroke Unit? = Yes

Q28 Were any of kinds of evidence **missing**? Please select all that apply.

- National Clinical Guidelines (1)
- Published research articles (7)
- A Pilot Project (8)
- Previous clinical experience with stroke patients (9)
- Anecdotal stories (10)
- Other, please specify (11) __________________________________________________
- Don't know (12)

Display This Question:

If Do you use the Free Water Protocol (FWP) in your Acute Stroke Unit? = No

Q29 What are the **sources of evidence** for use of the FWP in acute stroke patients? Please select all that apply.

- National Clinical Guidelines (1)
- Published research articles (4)
- A pilot project (5)
- Previous clinical experience (6)
- Anecdotal stories (7)
- Other, please specify (8) __________________________________________________
- None of the above (3)
- Don't know (2)

End of Block: Outer Setting

Start of Block: Inner setting

Display This Question:

If Do you use the Free Water Protocol (FWP) in your Acute Stroke Unit? = Yes

| 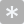 |
| --- |

Q30 Consider the following **work place** factors. To what extent do you agree that they affect implementation of the FWP in your Acute Stroke Unit?

|  | Strongly disagree (1) | Somewhat disagree (2) | Somewhat agree (3) | Strongly agree (4) |
| --- | --- | --- | --- | --- |
| Regular monitoring of patients (12) |  |  |  |  |
| Time intensity of acute stroke care (13) |  |  |  |  |
| Heavy workload (14) |  |  |  |  |
| Staff turnover (15) |  |  |  |  |
| Other, please specify (9) |  |  |  |  |

Display This Question:

If Do you use the Free Water Protocol (FWP) in your Acute Stroke Unit? = No

| 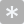 |
| --- |

Q31 Consider the following **work place** factors. To what extent do you agree that they would affect implementation of the FWP in your Acute Stroke Unit?

|  | Strongly disagree (1) | Somewhat disagree (2) | Somewhat agree (3) | Strongly agree (4) |
| --- | --- | --- | --- | --- |
| Regular monitoring of patients (1) |  |  |  |  |
| Time intensity of acute stroke care (2) |  |  |  |  |
| Heavy workload (3) |  |  |  |  |
| Staff turnover (4) |  |  |  |  |
| Other, please specify (5) |  |  |  |  |

Display This Question:

If Do you use the Free Water Protocol (FWP) in your Acute Stroke Unit? = Yes

| 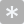 |
| --- |

Q32 Consider the following **work relationship** factors. To what extent do you agree that they affect implementation of the FWP in your Acute Stroke Unit?

|  | Strongly disagree (1) | Somewhat disagree (2) | Somewhat agree (3) | Strongly agree (4) |
| --- | --- | --- | --- | --- |
| Relationships with colleagues in your acute stroke unit (1) |  |  |  |  |
| Relationships with colleagues in other areas of the hospital (4) |  |  |  |  |
| Being part of the stroke multidisciplinary team (MDT) (5) |  |  |  |  |
| Cohesiveness of the stroke MDT (6) |  |  |  |  |
| Other, please specify (3) |  |  |  |  |

Display This Question:

If Do you use the Free Water Protocol (FWP) in your Acute Stroke Unit? = No

| 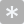 |
| --- |

Q33 Consider the following **work relationship** factors. To what extent do you agree that they would they affect implementation of the FWP in your Acute Stroke Unit?

|  | Strongly disagree (1) | Somewhat disagree (2) | Somewhat agree (3) | Strongly agree (4) |
| --- | --- | --- | --- | --- |
| Relationships with colleagues in your acute stroke unit (1) |  |  |  |  |
| Relationships with colleagues in other areas of the hospital (2) |  |  |  |  |
| Being part of the stroke multidisciplinary team (MDT) (3) |  |  |  |  |
| Cohesiveness of the stroke MDT (4) |  |  |  |  |
| Other, please specify (5) |  |  |  |  |

| 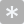 |
| --- |

Q34 How useful are the following ways for sharing information **within** your Acute Stroke Unit?

|  | Not very useful (2) | Slightly useful (3) | Moderately useful (4) | Very useful (5) |
| --- | --- | --- | --- | --- |
| Verbally at daily MDT meetings (1) |  |  |  |  |
| Verbally at weekly MDT meetings (4) |  |  |  |  |
| Email (5) |  |  |  |  |
| Electronic/ paper handover (6) |  |  |  |  |
| Medical notes (7) |  |  |  |  |
| Informally through word of mouth (8) |  |  |  |  |
| Other, please specify (9) |  |  |  |  |

| 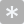 |
| --- |

Q35 How useful are the following ways for sharing information **across** the hospital?

|  | Not very useful (2) | Slightly useful (3) | Moderately useful (4) | Very useful (5) |
| --- | --- | --- | --- | --- |
| Hospital intranet (1) |  |  |  |  |
| Email (9) |  |  |  |  |
| Webinars (10) |  |  |  |  |
| Informally through word of mouth (11) |  |  |  |  |
| Other, please specify (12) |  |  |  |  |

Q36 The following **cultural factors** have been identified as factors affecting implementation of the FWP. Please select any that are applicable to your acute stroke unit.

- Clinicians’ expectations of learning new processes and procedures (1)
- Clinicians’ attitude to risk (4)
- Clinicians’ beliefs influencing clinical decision making (5)
- Clinicians’ previous experience influencing clinical decision making (6)
- Other, please specify (8) __________________________________________________

Q37Offering the Free Water Protocol is basic nursing care'. Do you agree with this statement?

- Yes (1)
- No (2)

| 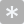 |
| --- |

Q38 The following factors have been identified as **barriers** to implementing the FWP in the Acute Stroke Unit setting. Please rate how significant they are as barriers.

|  | Not very significant (1) | Slightly significant (2) | Moderately significant (3) | Very significant (4) |
| --- | --- | --- | --- | --- |
| Fast pace of setting (1) |  |  |  |  |
| High turnover of patients (4) |  |  |  |  |
| Caseload demands (5) |  |  |  |  |
| Nurses availability (6) |  |  |  |  |
| Delegation of tasks for dependent patients (7) |  |  |  |  |
| Limited resources to record fluid intake (8) |  |  |  |  |
| Other, please specify (3) |  |  |  |  |

Display This Question:

If Do you use the Free Water Protocol (FWP) in your Acute Stroke Unit? = Yes

Q39 Are other duties **prioritised** over providing the FWP?

- Yes (1)
- No (2)

Display This Question:

If Do you use the Free Water Protocol (FWP) in your Acute Stroke Unit? = No

Q40 Do you think other duties would be **prioritised** over the providing the FWP?

- Yes (1)
- No (2)

Display This Question:

If Do you use the Free Water Protocol (FWP) in your Acute Stroke Unit? = Yes

Q41 On your Acute Stroke Unit **who** receives **training** about the FWP? Please select all that apply.

- Dietitians (6)
- Doctors (5)
- Families and carers (1)
- Nursing staff (4)
- Speech and Language Therapists (9)
- Support workers (7)
- Other, please specify. (8) __________________________________________________
- None of the above (10)
- Don't know (11)

Display This Question:

If Do you use the Free Water Protocol (FWP) in your Acute Stroke Unit? = Yes

And On your Acute Stroke Unit who receives training about the FWP? Please select all that apply. != None of the above

Q42 On your Acute Stroke Unit who provides **training** about the FWP? Please select all that apply.

- Speech and Language Therapists (1)
- Stroke Nurse Clinical Educator (3)
- Nursing staff (4)
- Other, please specify (5) __________________________________________________
- Don't know (6)

Display This Question:

If Do you use the Free Water Protocol (FWP) in your Acute Stroke Unit? = No

Or Do you use the Free Water Protocol (FWP) in your Acute Stroke Unit? = Yes

And On your Acute Stroke Unit who receives training about the FWP? Please select all that apply. = None of the above

Q43 On your Acute Stroke Unit **who** should be trained about the FWP? Please select all that apply.

- Dietitians (6)
- Doctors (5)
- Families and carers (1)
- Nursing staff (4)
- Speech and Language Therapists (9)
- Support workers (7)
- Other, please specify. (8) __________________________________________________
- Don't know (10)

Display This Question:

If Do you use the Free Water Protocol (FWP) in your Acute Stroke Unit? = No

Or Do you use the Free Water Protocol (FWP) in your Acute Stroke Unit? = Yes

And On your Acute Stroke Unit who receives training about the FWP? Please select all that apply. = None of the above

Q44 On your Acute Stroke Unit who should provide **training** about the FWP? Please select all that apply.

- Speech and Language Therapists (1)
- Stroke Nurse Clinical Educator (3)
- Nursing staff (4)
- Other, please specify (5) __________________________________________________

Display This Question:

If Do you use the Free Water Protocol (FWP) in your Acute Stroke Unit? = Yes

And On your Acute Stroke Unit who receives training about the FWP? Please select all that apply. != None of the above

Q45 What **types** **of training** are provided about the FWP in your Acute Stroke Unit? Please select all that apply.

- Group face-to-face teaching (1)
- Individual face-to-face teaching (3)
- Practical demonstration (4)
- Teach back (i.e. trainee explaining back to the trainer what they have understood from the session) (7)
- Written handouts (5)
- Peer support and modelling (6)
- Competency check (i.e. quiz) (8)
- E learning modules e.g. In house oral care training, Mouth Care Matters (9)
- Other, please specify (10) __________________________________________________

Display This Question:

If Do you use the Free Water Protocol (FWP) in your Acute Stroke Unit? = No

Or Do you use the Free Water Protocol (FWP) in your Acute Stroke Unit? = Yes

And On your Acute Stroke Unit who receives training about the FWP? Please select all that apply. = None of the above

Q46 What **types** **of training** about the FWP do you think should be given? Please select all that apply.

- Group face-to-face teaching (1)
- Individual face-to-face teaching (3)
- Practical demonstration (4)
- Teach back (i.e. trainee explaining back to the trainer what they have understood from the session) (7)
- Written handouts (5)
- Peer support and modelling (6)
- Competency check (i.e. quiz) (8)
- E learning modules e.g. In house oral care training, Mouth Care Matters (9)
- Other, please specify (10) __________________________________________________

Display This Question:

If Do you use the Free Water Protocol (FWP) in your Acute Stroke Unit? = Yes

Q47 The following factors have been identified as **barriers** to implementing the FWP. Please indicate if these apply to your experience.

- Daily shift changes impacting on ability to train ward staff (1)
- Organisation required to deliver ongoing training (7)
- Time required to deliver ongoing training (4)
- No clear written protocol (5)
- Other, please specify (3) __________________________________________________
- None of the above (6)

Display This Question:

If Do you use the Free Water Protocol (FWP) in your Acute Stroke Unit? = No

Q48 The following factors have been identified as **barriers** to implementation of the FWP. Please indicate if these would apply to your Acute Stroke Unit.

- Ongoing training requirements due to daily shift changes (1)
- Organisation required to deliver ongoing training (6)
- Time required to deliver ongoing training (4)
- No clear written protocol (5)
- Other, please specify (3) __________________________________________________

End of Block: Inner setting

Start of Block: Individual Characteristics

| 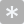 |
| --- |

Q49 To what extent do you agree that the following **patient outcomes** are **facilitators** to the implementation of the FWP?

|  | Strongly disagree (1) | Somewhat disagree (2) | Somewhat agree (3) | Strongly agree (4) |
| --- | --- | --- | --- | --- |
| Patient comfort (7) |  |  |  |  |
| Normalisation (i.e. being offered water along with other patients) (12) |  |  |  |  |
| Patient choice (13) |  |  |  |  |
| Improved hydration (14) |  |  |  |  |
| Other, please specify (8) |  |  |  |  |

| 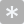 |
| --- |

Q50 To what extent do you agree that the following **patient outcomes** are **barriers** to the implementation of the FWP?

|  | Strongly disagree (1) | Somewhat disagree (2) | Somewhat agree (3) | Strongly agree (4) |
| --- | --- | --- | --- | --- |
| Aspiration (17) |  |  |  |  |
| Development of chest complications (15) |  |  |  |  |
| Other, please specify (12) |  |  |  |  |

Q51 In your opinion does the potential for negative outcomes **outweigh** the potential positive outcomes for patients of implementing the FWP?

- Always (1)
- Sometimes (2)
- Never (3)

Display This Question:

If Do you use the Free Water Protocol (FWP) in your Acute Stroke Unit? = Yes

| 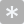 |
| --- |

Q52 The following factors have been identified as influencing the success of the FWP. Please indicate the extent to which you think they are helpful to the delivery of the FWP.

|  | Not very helpful (1) | Slightly helpful (2) | Moderately helpful (3) | Very helpful (4) |
| --- | --- | --- | --- | --- |
| Having knowledge of the FWP (1) |  |  |  |  |
| Access to clinicians with high degree of dysphagia expertise (2) |  |  |  |  |
| Lack of clear instructions (3) |  |  |  |  |
| Experience of the FWP (4) |  |  |  |  |
| Knowledge and experience working with the stroke population (5) |  |  |  |  |
| Incomplete documentation (6) |  |  |  |  |
| Assumptions about stroke presentations (7) |  |  |  |  |
| Sporadic use of the FWP (8) |  |  |  |  |
| Lack of awareness of the FWP from other professionals (9) |  |  |  |  |
| Lack of mouth care skills (10) |  |  |  |  |
| Misinterpretation of protocol by family (11) |  |  |  |  |
| Staff forgetting to offer water (12) |  |  |  |  |
| Nurses not following the protocol (13) |  |  |  |  |
| Sufficient staff resource to position patients (14) |  |  |  |  |
| Sufficient staff resource to supervise patients drinking water (15) |  |  |  |  |
| Sufficient staff resource to provide mouth care (16) |  |  |  |  |
| Other, please specify (17) |  |  |  |  |

Display This Question:

If Do you use the Free Water Protocol (FWP) in your Acute Stroke Unit? = No

| 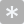 |
| --- |

Q53 The following factors have been identified as influencing the success of the FWP. Please indicate the extent to which you think they could be helpful to the delivery of the FWP.

|  | Not very helpful (1) | Slightly helpful (2) | Moderately helpful (3) | Very helpful (4) |
| --- | --- | --- | --- | --- |
| Having knowledge of the FWP (1) |  |  |  |  |
| Access to clinicians with high degree of dysphagia expertise (2) |  |  |  |  |
| Lack of clear instructions (3) |  |  |  |  |
| Experience of the FWP (4) |  |  |  |  |
| Knowledge and experience working with the stroke population (5) |  |  |  |  |
| Incomplete documentation (6) |  |  |  |  |
| Assumptions about stroke presentations (7) |  |  |  |  |
| Sporadic use of the FWP (8) |  |  |  |  |
| Lack of awareness of the FWP from other professionals (9) |  |  |  |  |
| Lack of mouth care skills (10) |  |  |  |  |
| Misinterpretation of protocol by family (11) |  |  |  |  |
| Staff forgetting to offer water (12) |  |  |  |  |
| Nurses not following the protocol as intended (13) |  |  |  |  |
| Sufficient staff resource to position patients (14) |  |  |  |  |
| Sufficient staff resource to supervise patients drinking water (15) |  |  |  |  |
| Sufficient staff resource to provide mouth care (16) |  |  |  |  |
| Other, please specify (17) |  |  |  |  |

Display This Question:

If Do you use the Free Water Protocol (FWP) in your Acute Stroke Unit? = Yes

Q54 Does involvement in implementing the FWP **fit** within your **current role**?

- Yes (2)
- No (3)

Display This Question:

If Do you use the Free Water Protocol (FWP) in your Acute Stroke Unit? = No

Q55 Would involvement in implementing the FWP **fit** within your **current role**?

- Yes (2)
- No (3)

Display This Question:

If Do you use the Free Water Protocol (FWP) in your Acute Stroke Unit? = Yes

Q56 Do you **involve families** and **informal caregivers** to support the delivery of the FWP?

- Yes (1)
- No (2)

Display This Question:

If Do you use the Free Water Protocol (FWP) in your Acute Stroke Unit? = No

Q57 Would you **involve families** and **informal caregivers** to support the delivery of the FWP?

- Yes (1)
- No (2)

Display This Question:

If Do you use the Free Water Protocol (FWP) in your Acute Stroke Unit? = Yes

And Do you involve families and informal caregivers to support the delivery of the FWP? = Yes

Q58 **What support** do families and informal caregivers provide? Please select all that apply.

- Positioning (1)
- Mouth care (4)
- Offering water (5)
- Supervision while drinking (6)
- Other, please specify (7) __________________________________________________
- None of the above (8)

Display This Question:

If Do you use the Free Water Protocol (FWP) in your Acute Stroke Unit? = No

And Would you involve families and informal caregivers to support the delivery of the FWP? = Yes

Q59 What support should families and informal caregivers provide? Please select all that apply.

- Positioning (1)
- Mouth care (4)
- Offering water (5)
- Supervision while drinking (6)
- Other, please specify (7) __________________________________________________
- None of the above (8)

| 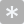 |
| --- |

Q60 The following factors have been identified as affecting individuals’ **motivation to engage** with the FWP. In your opinion, please indicate how important these factors are.

|  | Not very important (1) | Slightly important (2) | Moderately important (3) | Very Important (4) |
| --- | --- | --- | --- | --- |
| Patient choice (21) |  |  |  |  |
| Patient desire to drink water (26) |  |  |  |  |
| Staff uncertainty about legal liability (27) |  |  |  |  |
| Negative staff attitudes around completing mouthcare (28) |  |  |  |  |
| SLT time taken to set up the FWP (29) |  |  |  |  |
| SLT uncertainty that the FWP would be implemented as intended (30) |  |  |  |  |
| Nurses desire to make time for the FWP (31) |  |  |  |  |
| Other, please specify (22) |  |  |  |  |

End of Block: Individual Characteristics

Start of Block: Implementation Process

Display This Question:

If Do you use the Free Water Protocol (FWP) in your Acute Stroke Unit? = Yes

| 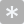 |
| --- |

Q61 How important are the following **team working** factors in implementing the FWP?

|  | Not very important (1) | Slightly important (2) | Moderately important (3) | Very important (4) |
| --- | --- | --- | --- | --- |
| Dedicated implementation leaders and champions (4) |  |  |  |  |
| Leadership by senior clinicians (9) |  |  |  |  |
| Team support to identify suitable patients (10) |  |  |  |  |
| Communication within the stroke MDT (11) |  |  |  |  |
| Team approach to monitoring patients (12) |  |  |  |  |
| Availability of family support (13) |  |  |  |  |
| Other, please specify (5) |  |  |  |  |

Display This Question:

If Do you use the Free Water Protocol (FWP) in your Acute Stroke Unit? = No

| 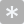 |
| --- |

Q62 How important would the following **team working** factors be in implementing the FWP?

|  | Not very important (1) | Slightly important (2) | Moderately important (3) | Very important (4) |
| --- | --- | --- | --- | --- |
| Dedicated implementation leaders and champions (4) |  |  |  |  |
| Leadership by senior clinicians (9) |  |  |  |  |
| Team support to identify suitable patients (10) |  |  |  |  |
| Communication within the stroke MDT (11) |  |  |  |  |
| Team approach to monitoring patients (12) |  |  |  |  |
| Availability of family support (13) |  |  |  |  |
| Other, please specify (5) |  |  |  |  |

Q63 The FWP is considered by some as the sole domain of the speech and language therapist. Please indicate you see this as an advantage or disadvantage?

- Advantage (1)
- Disadvantage (2)

Display This Question:

If Do you use the Free Water Protocol (FWP) in your Acute Stroke Unit? = Yes

Q64 The following **strategies** have been used or recommended to implement the FWP in the Acute Stroke Unit setting. Which if any have you used in your Acute Stroke Unit? Please select all that apply.

- Having an established mouth care protocol before introducing the FWP to your acute stroke unit (4)
- Standard operating procedure for FWP (5)
- Written instructions (6)
- Sheet at patient’s bedside to monitor mouth care (7)
- Sheet at patient’s bedside for staff and caregivers to measure water intake (8)
- Clear communication between MDT to monitor patient status (9)
- Regular communication between MDT at handover (10)
- Rolling education program to team members on the rules risks and benefits of the FWP (11)
- Education to family (12)
- Nurses offering water in lieu of thickened fluids between meals (13)
- Daily monitoring of symptoms of potential signs of deterioration associated with aspiration (14)
- Education to patient (15)
- Outlining clearly defined roles and responsibilities for each discipline involved in providing the FWP (16)
- Individualised patient care plan (17)
- Individual’s roles clearly identified on patient’s care plan (18)
- Identification of a FWP champion (19)
- Leadership and modelling of decision making and implementation (20)
- Involving patients in the decision making process (21)
- Other, please specify (22) __________________________________________________
- None of the above (23)

Display This Question:

If Do you use the Free Water Protocol (FWP) in your Acute Stroke Unit? = No

Q65 The following **strategies** have been used or recommended to implement the FWP in the Acute Stroke Unit setting. Which if any do you think would be important in your Acute Stroke Unit? Please select all that apply.

- Having an established mouth care protocol before introducing the FWP to your acute stroke unit (4)
- Standard operating procedure for FWP (5)
- Written instructions (6)
- Sheet at patient’s bedside to monitor mouth care (7)
- Sheet at patient’s bedside for staff and caregivers to measure water intake (8)
- Clear communication between MDT to monitor patient status (9)
- Regular communication between MDT at handover (10)
- Rolling education program to team members on the rules risks and benefits of the FWP (11)
- Education to family (12)
- Nurses offering water in lieu of thickened fluids between meals (13)
- Daily monitoring of symptoms of potential signs of deterioration associated with aspiration (14)
- Education to patient (15)
- Outlining clearly defined roles and responsibilities for each discipline involved in providing the FWP (16)
- Individualised patient care plan (17)
- Individual’s roles clearly identified on patient’s care plan (18)
- Identification of a FWP champion (19)
- Leadership and modelling of decision making and implementation (20)
- Involving patients in the decision making process (21)
- Other, please specify (22) __________________________________________________
- None of the above (23)

Q66 Which of these would be important to include in a patient’s FWP **care plan**? Please select all that apply.

- Patient or consultee consent for following the FWP (1)
- Accountability for mouth care (4)
- Evidence that mouth care has been provided (5)
- Evidence of education for staff (6)
- Evidence of education for family (7)
- Level of supervision required for the patient (8)
- Swallowing strategies (9)
- Evidence that water has been offered (10)
- Individual fluid requirements (including fluid restrictions) (11)
- Other, please specify (12) __________________________________________________
- None of the above (13)

End of Block: Implementation Process

Start of Block: Closing questions

Q67 The following question gives you the opportunity to tell us anything more about the barriers and facilitators to implementing the FWP in the acute stroke unit setting.

________________________________________________________________

Display This Question:

If Do you use the Free Water Protocol (FWP) in your Acute Stroke Unit? = Yes

Q68 Please let us know if you would be happy to share your hospital documentation about the FWP.

- Yes (4)
- No (5)

Q69 Please let us know if you would like the research team to send you the results of this research.

- Yes (1)
- No (2)

Display This Question:

If Do you use the Free Water Protocol (FWP) in your Acute Stroke Unit? = Yes

And Please let us know if you would be happy to share your hospital documentation about the FWP. = Yes

Or Please let us know if you would like the research team to send you the results of this research. = Yes

Or Do you use the Free Water Protocol (FWP) in your Acute Stroke Unit? = No

And Please let us know if you would like the research team to send you the results of this research. = Yes

Q70 Please provide your name and email address for us to contact you.

________________________________________________________________

End of Block: Closing questions

**3: Advertising material for staff survey**

**Social Media Post**

**1^st^ post**

Are you a UK stroke professional based in a hospital Acute Stroke Unit working with stroke survivors with #dysphagia? We would like to hear what you think are the barriers and facilitators of implementing the #FreeWaterProtocol in an Acute Stroke Unit.

Please click here to complete the survey: [insert hyperlink].

Upload powerpoint slide with QR code.

**Reminder 1**

**UK Stroke Professionals:**

Have you completed our survey yet about the barriers and facilitators of implementing the #FreeWaterProtocol in an Acute Stroke Unit?

If you are a Speech and Language Therapist (SLT), nurse, dietitian, doctor or clinical support staff, we want to hear from you!

To take part, please click here for the survey: [insert hyperlink]

**Closing date: [xx/xx/xx]**

Upload powerpoint with QR code.

**Reminder 2**

**UK Stroke Professionals**:

Last chance to complete our survey about the barriers and facilitators of implementing the #FreeWaterProtocol in an Acute Stroke Unit setting and to shape this important Stroke Association research!

If you are a Speech and Language Therapist (SLT), nurse, dietitian, doctor or clinical support staff, we want to hear from you!

To take part, please click here for the survey: [insert hyperlink]

**Closing date: [insert date]**

Upload powerpoint with QR code.

**Email message**

Dear [Recipient],

I am writing to ask for your help with an important survey I am conducting about the barriers and facilitators to the implementation of the **Free Water Protocol** in the acute stroke unit setting.

If your Acute Stroke Unit uses the Free Water Protocol, I am interested to hear about your experiences of implementing the protocol. If your Acute Stroke Unit does not routinely use the Free Water Protocol, I am interested in your perceptions of the barriers and facilitators to its implementation.

This research is being funded by the Stroke Association, and your responses will help shape our understanding of the feasibility of implementing the Free Water Protocol in Acute Stroke Units. I hope you can spend a few minutes sharing what happens in your hospital Acute Stroke Unit.

**To participate, please click here for the survey: [insert hyperlink]. We estimate it will take about fifteen minutes to complete the questionnaire.**

**Please cascade this survey to your stroke multidisciplinary team**. We would love to hear from speech and language therapists, nurses, dietitians, doctors, and clinical support staff who may be involved in the implementation of the Free Water Protocol.

The survey is anonymous, and your answers will not be linked with your department in any reports of the data. Participation is voluntary. Should you have any questions or comments, please contact me at sabrina.eltringham@nhs.net or Nicola Martindale at nicola.martindale@nhs.net.

The closing date for the survey is [Insert date].

I very much appreciate your help with this study.

Many thanks,

Sabrina Eltringham

**Email reminder**

Dear [Recipient],

Recently, I sent you an email asking for your help with an important survey. The survey is part of a Stroke Association research study to learn more about the **feasibility of implementing the Free Water Protocol in the Acute Stroke Unit setting**.

If you have already completed the survey, thank you for contributing to this research.

If you haven’t, this is a reminder that the **closing date for the survey is [xx/xx/xx].** It should only take a few minutes to complete the questionnaire.

Simply click on this link and you will automatically be logged into the survey: [insert hyperlink]

The results of this study will help better understand the barriers and facilitators for implementing the Free Water Protocol in the acute stroke unit setting. Your participation is very important, and I appreciate you considering this request.

Best wishes,

Sabrina Eltringham

**Copy material for Professional Networks**

**Important Survey for [insert name of profession e.g., Nurses] in Acute Stroke**

Dear Colleagues,

Sabrina Eltringham, a Stroke Association Postdoctoral Research Fellow, is conducting a research study on an alternative to thickened fluids for stroke survivors with dysphagia.

The Free Water Protocol (FWP) allows individuals at risk of aspiration to drink unthickened water between meals, following a set of guidelines designed to maximize patient safety. The National Institute for Health and Care Excellence has recently recommended further research on the FWP due to its promising yet limited evidence base. It is also uncertain whether the FWP can be effectively implemented in acute stroke wards across the UK.

If your Acute Stroke Unit uses the Free Water Protocol, Sabrina would like to hear about your experiences with its implementation. If your unit does not routinely use the Free Water Protocol, she is interested in your perspectives on the potential barriers and facilitators to its adoption.

**Please take a moment to complete this quick survey to help shape this important research** [insert hyperlink].

Thank you for your time and contribution.

Best regards,

[Your Name]
